# Longitudinal spatial correlation of retinal sensitivity and structural changes in macular telangiectasia type 2

**DOI:** 10.1186/s40942-026-00873-8

**Published:** 2026-05-29

**Authors:** Seungmo Kim, Yoon Jeon Kim, Young Hee Yoon, Junyeop Lee

**Affiliations:** 1 Department of Ophthalmology, Veterans Health Service Medical Center, Seoul, Korea; 2 Department of Ophthalmology, Asan Medical Center, University of Ulsan College of Medicine, 388-1 Pungnap-2-dong, Songpa-gu, Seoul, 05505 Korea; 3 Graduate School of Health Science and Technology, UNIST, Ulsan, South Korea

**Keywords:** Macular telangiectasia type 2, MacTel, Microperimetry, Retinal sensitivity

## Abstract

**Purpose:**

This study aimed to investigate the spatial correlation between retinal structure and function in macular telangiectasia type 2 (MacTel) using Compass fundus-tracked microperimetry.

**Methods:**

Medical records of 54 eyes from 28 patients were retrospectively reviewed. The relationship between retinal sensitivity and structural parameters including retinal thickness, ellipsoid zone (EZ) integrity, and visual acuity was analyzed. Retinal sensitivity was calculated for nine macular sectors defined by the Early Treatment Diabetic Retinopathy Study (ETDRS) grid, and changes over a 2-year follow-up were evaluated.

**Results:**

The mean central retinal thickness was 254.3 ± 103.7 μm, and the mean central retinal sensitivity (mRS) was 28.9 ± 5.4 dB. Microscotomas (< 10 dB) were detected in 18.5% of eyes, predominantly in the temporal macula. Although visual acuity and retinal thickness remained stable during follow-up, the central mRS showed a significant decline (29.51 ± 2.02 dB vs. 28.84 ± 1.38 dB, *p* = 0.038). The spatial distribution of microscotomas corresponded closely to areas of EZ loss, although pointwise mismatch occasionally occurred.

**Conclusion:**

These findings demonstrate that Compass microperimetry can detect subtle longitudinal functional decline despite a lack of significant change in visual acuity and retinal thickness. These results suggest that microperimetry may serve as a complementary functional biomarker for early disease monitoring in MacTel.

## Key messages

**What is known:**

In macular telangiectasia type 2, structural alterations such as temporal parafoveal thinning and ellipsoid zone disruption are associated with localized loss of retinal sensitivity.

**What is new:**

Central mean retinal sensitivity showed a longitudinal decline despite stable retinal thickness and best-corrected visual acuity over two years, suggesting that fundus-tracked microperimetry may provide complementary functional information in early MacTel. Fundus-tracked microperimetry and central 4-point mean retinal sensitivity may serve as a feasible functional endpoint for upcoming interventional studies in macular telangiectasia type 2.

## Introduction

Macular telangiectasia type 2 (MacTel) is a bilateral, slowly progressive neurodegenerative disorder that primarily affects the parafoveal retina, typically initiating temporally to the foveal center [1, 2]. Clinically, it is characterized by loss of retinal transparency, crystalline deposits, capillary dilation, foveal thinning, and, in advanced stages, neovascular proliferation, often leading to a decline in visual acuity to 20/40 or worse [3–5]. Although previously regarded as a vascular disease, increasing evidence supports the concept of primary Müller cell dysfunction as a central pathogenic mechanism, leading to secondary photoreceptor loss and vascular remodeling [6]. However, the mechanisms underlying the spatial confinement of retinal pigment epithelium (RPE) and subretinal abnormalities within the MacTel zone remain poorly understood.

Recent advances in multimodal imaging have enabled simultaneous assessment of retinal morphology and localized function [7, 8]. Fundus-tracked microperimetry allows quantitative evaluation of retinal sensitivity while correcting for fixation instability, providing a valuable tool for structure–function correlation studies [9, 10]. Earlier devices such as the MP-1 were limited by narrow dynamic range and ceiling effects, whereas the Compass microperimeter (CenterVue, Italy) offers a wider dynamic range of 36 dB and improved reproducibility through active fundus tracking [11].

Prior research within the MacTel Project framework has established the clinical relevance of this structure-function paradigm. MP-1 microperimetry studies demonstrated that scotomas are often localized to the temporal parafoveal MacTel zone, while longitudinal analyses showed that ellipsoid zone (EZ) loss is associated with functional loss over time [12, 13]. These findings support EZ disruption as a key structural marker linked to localized visual dysfunction. However, most existing studies have relied on MP-1-based testing or trial-specific analyses. Data utilizing Compass fundus-tracked microperimetry, particularly with sectoral ETDRS-based spatial mapping to bridge structural thinning and functional loss, remain limited.

The clinical significance of microperimetry has been further emphasized by the recent approval of ENCELTO™ (revakinagene taroretcel-lwey), an encapsulated cell-based gene therapy delivering ciliary neurotrophic factor, representing the first cell-based neuroprotective treatment for any neurodegenerative retinal or central nervous system disease [14, 15]. In pivotal ENCELTO trials, while the rate of EZ loss served as the primary endpoint, microperimetry was utilized as a secondary functional endpoint to assess therapeutic efficacy, validating its role as a sensitive biomarker of localized visual function [16]. Elucidating the spatial relationship between localized structural damage and retinal sensitivity in MacTel is essential for interpreting microperimetry-based outcomes and designing future therapeutic trials. This study therefore aimed to investigate the topographic correlation between retinal structure and function in patients with MacTel type 2 using Compass fundus-tracked microperimetry.

## Methods

### Study design

This retrospective study reviewed medical records of patients diagnosed with macular telangiectasia type 2 (MacTel) at Asan Medical Center between March 2020 and May 2023. The study protocol was approved by the Institutional Review Board of Asan Medical Center and adhered to the tenets of the Declaration of Helsinki. Given the retrospective design, the requirement for informed consent was waived.

### Participants

Patients were included if they had at least one gradable microperimetry examination performed using the Compass microperimeter (CenterVue, Italy). All participants underwent comprehensive ophthalmic examinations at baseline and 12-month follow-up visits, including best-corrected visual acuity (BCVA), intraocular pressure measurement, slit-lamp biomicroscopy, and fundus photography. Spectral-domain optical coherence tomography (SD-OCT) images were acquired using a Spectralis OCT system (Heidelberg Engineering, Heidelberg, Germany). Macular volume scans consisted of 49 horizontal B-scans covering the central area 20° × 20° centered on the fovea. Images with signal strength ≥ 6 were included. Automated layer segmentation, specifically the boundaries between the internal limiting membrane (ILM) and the retinal pigment epithelium (RPE), was manually verified by a single examiner (S.K) to ensure accurate thickness measurements. Central retinal thickness (CRT) was defined as the mean retinal thickness within a 1-mm diameter circle centered on the fovea. Additionally, the qualitative integrity of the external limiting membrane (ELM) and the ellipsoid zone (EZ) was assessed. EZ status was categorized as intact, irregular or loss based on the continuity and reflectivity of the EZ band on foveal and parafoveal B-scans. For longitudinal structural assessment, baseline and final-visit OCT scans were qualitatively reviewed for changes in EZ integrity. Exclusion criteria included (1) coexisting retinal diseases such as diabetic retinopathy, age-related macular degeneration, retinal vascular occlusion, epiretinal membrane, or uveitis; (2) glaucoma or dense cataract interfering with visual function; (3) spherical equivalent > ± 6.0 diopters; and (4) poor-quality imaging or unreliable examinations.

### Diagnosis of macular telangiectasia type 2

MacTel type 2 was diagnosed by retinal specialists based on characteristic clinical and multimodal imaging findings. Diagnostic features included parafoveal loss of retinal transparency, crystalline deposits, right-angled venules, and/or pigment plaques on color fundus photography, together with SD-OCT findings such as temporal parafoveal thinning, hyporeflective cavitations, and disruption of EZ or ELM. Fundus autofluorescence and fluorescein angiography, when available, were used as supportive imaging modalities.

### Microperimetry

**Fig. 1:**
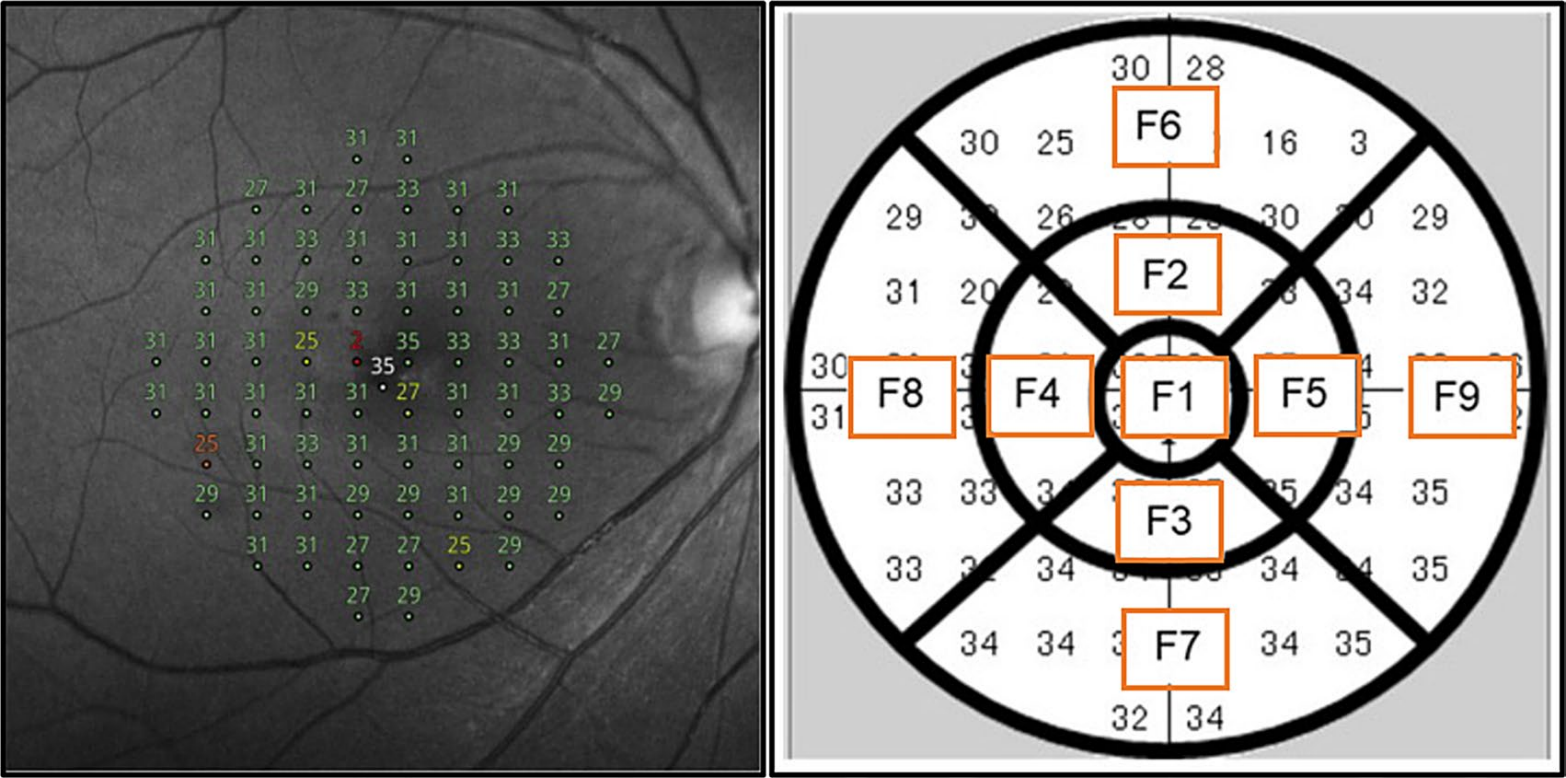
Measurement and spatial mapping of retinal sensitivity using Compass microperimetry. (Left) Representative fundus image showing pointwise retinal sensitivity measured with Compass microperimetry using a standard 10 − 2 grid (68 stimulus loci). Color-coded values represent threshold sensitivities in decibels (dB). (Right) Schematic of the Early Treatment Diabetic Retinopathy Study (ETDRS) grid used for spatial analysis. Each microperimetry test locus was assigned to one of nine ETDRS subfields (F1–F9), and the mean retinal sensitivity (mRS) was calculated as the arithmetic mean of all loci within each subfield, with the central mRS (F1) defined by the four test points nearest the fovea

**Fig. 2:**
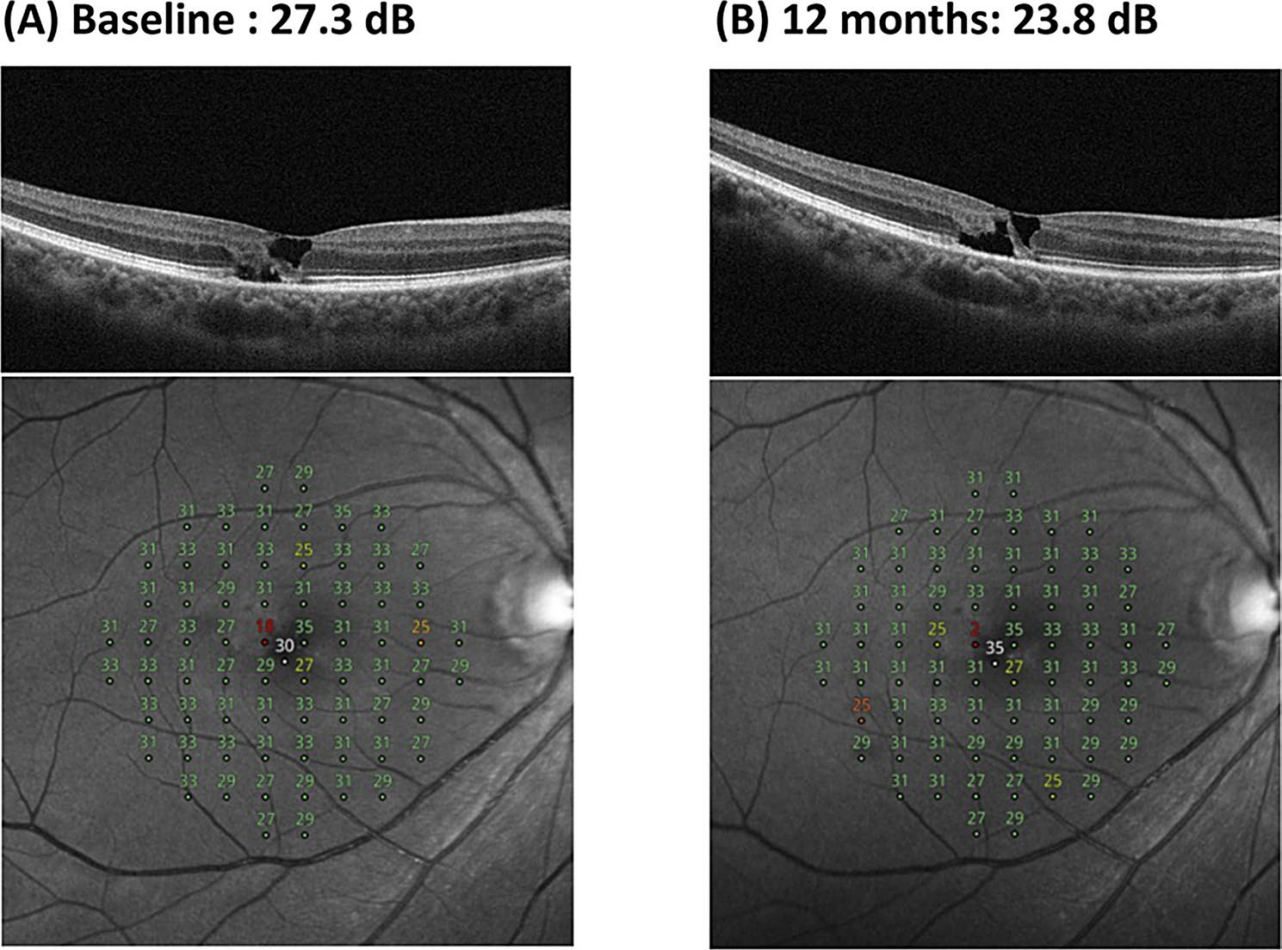
Representative case of macular telangiectasia type 2 showing structural and functional progression. A 70-year-old woman presented with decreased vision in her right eye. (**A**) At baseline, best-corrected visual acuity (BCVA) was 0.8. Spectral-domain optical coherence tomography (SD-OCT) revealed inner retinal cavitation with focal ellipsoid-zone (EZ) disruption. Compass microperimetry demonstrated a mean retinal sensitivity (mRS) of 27.3 dB in the four loci nearest the fovea. (**B**) At the 12-month follow-up, BCVA remained unchanged, but the area of EZ loss had expanded on SD-OCT. Corresponding microperimetry showed decreased central sensitivity (mRS = 23.8 dB) with spatial concordance to the region of structural disruption. This case illustrates that localized functional decline can occur despite preserved visual acuity, highlighting the sensitivity of microperimetry for detecting early disease progression in MacTel

Retinal sensitivity was measured using Compass microperimetry under mesopic conditions with a 10 − 2 grid comprising 68 loci. Visual field tests were deemed reliable when the false-positive rate was < 18% and the false-negative rate was < 33%. Sensitivity data were integrated into the Early Treatment Diabetic Retinopathy Study (ETDRS) grid to calculate mean retinal sensitivity (mRS) across nine macular sectors (Fig. 1). The ETDRS grid was centered on the fovea, and each Compass 10 − 2 test locus was assigned to the corresponding ETDRS subfield using the fundus-tracked retinal image. Alignment between the microperimetry image and OCT-based ETDRS grid was reviewed manually to ensure topographic correspondence. The topographic relationship between mRS and retinal structure on SD-OCT was analyzed at baseline and follow-up and a representative case is demonstrated in Fig. 2. Microscotomas were defined as loci with thresholds < 10 decibels (dB) [7, 13]. The spatial distribution of microscotomas was compared with corresponding anatomic changes on OCT.

### Statistical analysis

Continuous variables are presented as mean ± standard deviation. BCVA was converted to logarithm of the minimum angle of resolution (logMAR) for analysis. Linear mixed-effects models were employed to account for inter-eye correlation arising from the inclusion of both eyes from the same patient. For cross-sectional comparisons at baseline, EZ status was treated as a fixed effect, with patient ID as a random intercept. For longitudinal analyses of changes in BCVA, CRT, and mean retinal sensitivity (mRS), visit was defined as a fixed effect. Random intercepts were specified for patients and eyes nested within patients to account for both the correlation between fellow eyes and the repeated measurements within the same eye. A two-tailed *p* < 0.05 was considered statistically significant. All analyses were conducted using SPSS version 27.0 (IBM Corp., Armonk, NY, USA).

## Results

**Fig. 3:**
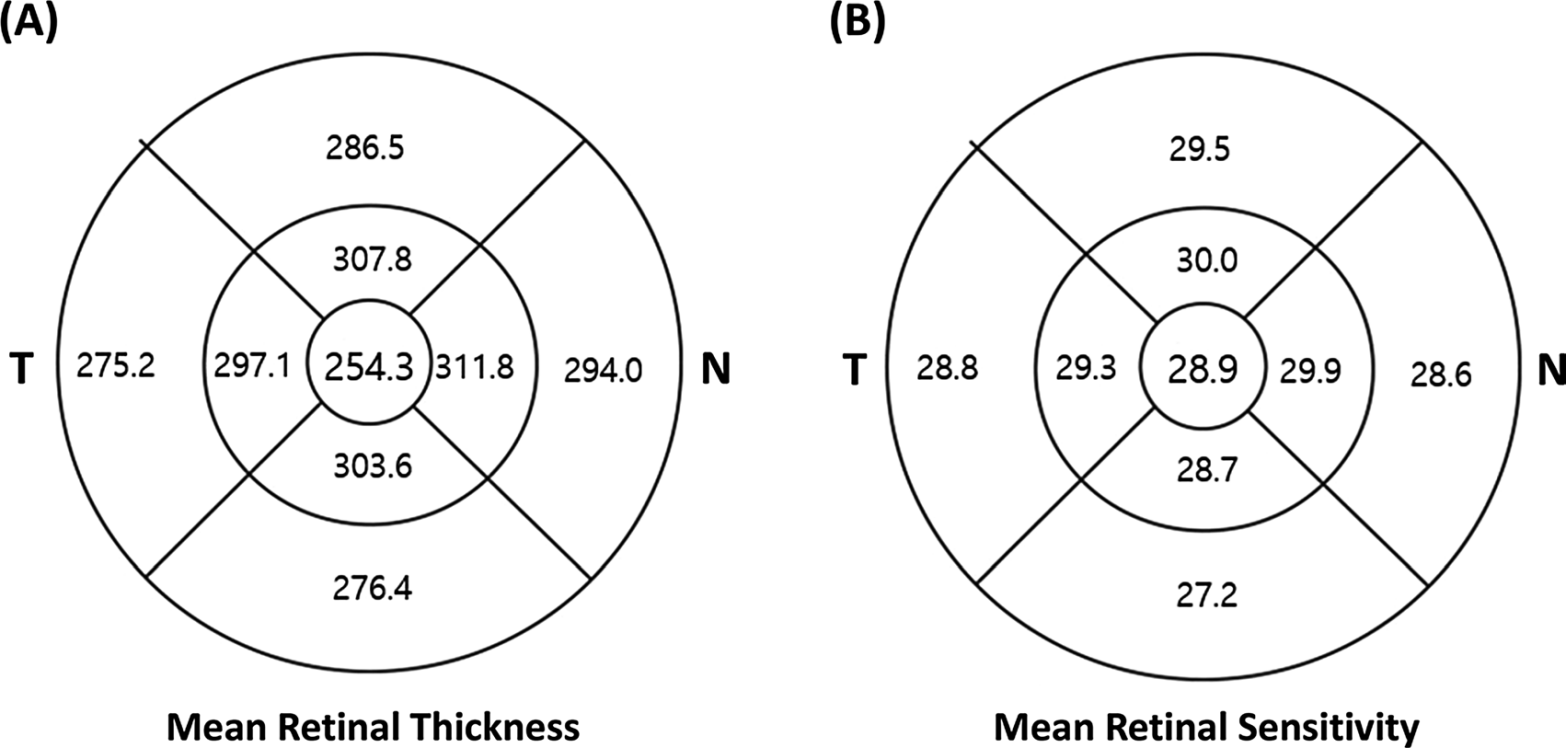
Mean retinal thickness and mean retinal sensitivity across ETDRS subfields in macular telangiectasia type 2. (**A**) Distribution of mean retinal thickness (µm) measured by spectral-domain OCT. The central subfield (F1) showed an average thickness of 254.3 μm, with temporal sectors generally thinner than nasal sectors. (**B**) Corresponding map of mean retinal sensitivity (dB) measured by Compass microperimetry. The central mean retinal sensitivity (mRS) was 28.9 dB, with relatively uniform values across parafoveal and perifoveal regions. These maps illustrate the spatial dissociation between mild structural thinning and largely preserved retinal sensitivity in early-stage MacTel

A total of 54 eyes from 28 patients with MacTel were analyzed. The mean age was 64.9 ± 15.3 years, and 78.6% were female. Baseline BCVA averaged logMAR 0.15 ± 0.10 (approx. 20/30 Snellen). Table 1 summarizes demographics and clinical characteristics. Retinal thickness and mean retinal sensitivity (mRS) across ETDRS grid sectors are shown in Fig. 3. The mean CRT (F1) was 254.3 ± 103.7 μm. Retinal thickness varied across subfields, with temporal sectors thinner than nasal ones (F8: 275.2 ± 119.7 μm, F4: 297.1 ± 93.5 μm vs. F9: 294.0 ± 72.5 μm, F5: 311.8 ± 98.5 μm). The mean retinal sensitivity of the central region (F1) was 28.9 ± 5.4 dB, with no significant inter-sectoral differences.

**Table 1:** Demographic and baseline clinical characteristics of patients with macular telangiectasia type 2

Parameters	Value	
Eyes/Patients (n)	54/28	
Age (y)	64.9 ± 15.3 (range, 34 ~ 81)	
Sex (male/female)	6/22 (female 78.6%)	
Spherical equivalent (Diopter)	-0.25 ± 1.31 (range, -3.0 ~ + 2.0)	
Baseline IOP (mmHg)	12.8 ± 1.69	
logMAR BCVA	0.15 ± 0.10	
**Mean retinal thickness** (um)		
F1	254.31 ± 103.70	
F2	307.84 ± 121.42	
F3	303.65 ± 81.98	
F4	297.15 ± 93.56	
F5	311.89 ± 98.59	
F6	286.54 ± 140.17	
F7	276.45 ± 131.81	
F8	275.21 ± 119.76	
F9	294.04 ± 72.52	
Mean MD (dB)	-0.31 ± 4.32	
Mean PSD (dB)	2.99 ± 3.75	
**Mean retinal sensitivity** (dB)		
F1	28.93 ± 5.47	
F2	30.08 ± 9.13	
F3	28.74 ± 8.79	
F4	29.37 ± 7.25	
F5	29.95 ± 6.82	
F6	29.56 ± 9.77	
F7	27.25 ± 8.18	
F8	28.86 ± 5.97	
F9	28.60 ± 8.85	
Number of microscotomas	10 (18.5%)	
**OCT characteristics**		
** ELM**		
Intact	33 (61.1%)	
Irregular	12 (22.2%)	
Loss	9 (16.7%)	
** Ellipsoid zone**		
Intact	33 (61.1%)	
Irregular	12 (22.2%)	
Loss	9 (16.7%)	

Data indicate the mean ± standard deviation

**Fig. 4:**
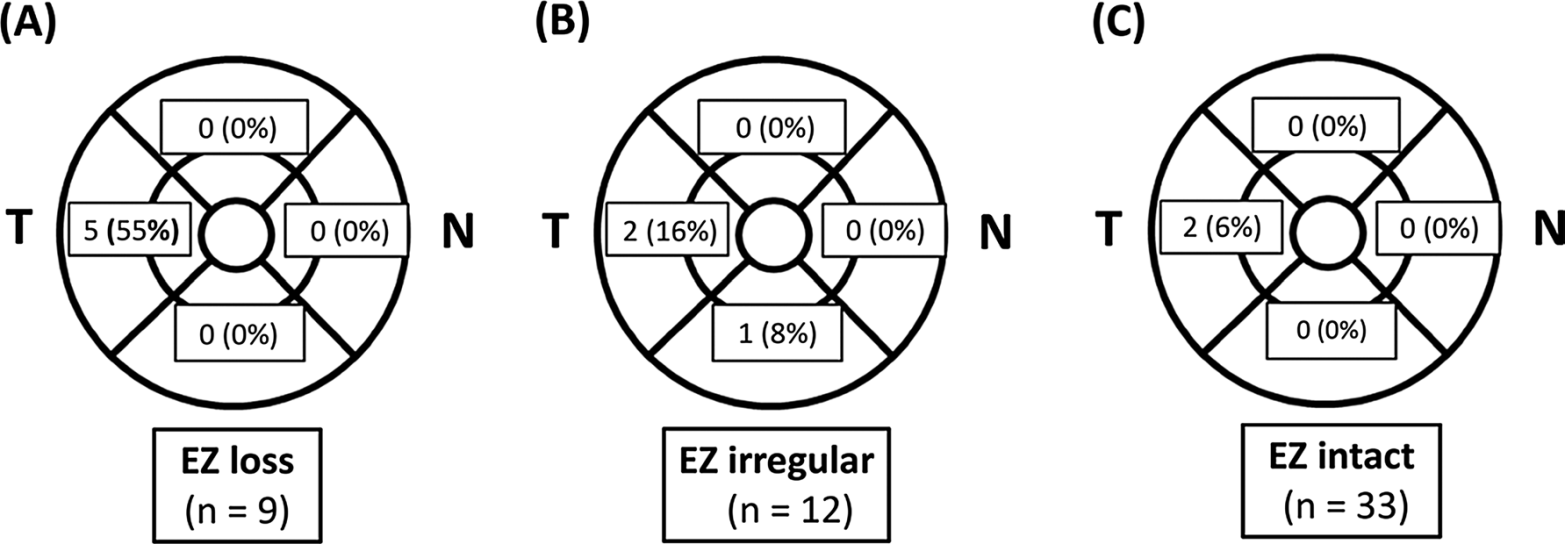
Spatial distribution of microscotomas according to ellipsoid zone (EZ) integrity. ETDRS subfield maps illustrate the frequency and percentage of eyes with microscotomas (threshold < 10 dB) within each macular sector, stratified by EZ status. (**A**) Eyes with EZ loss (*n* = 9) demonstrated a high prevalence of temporally located microscotomas (5 eyes, 55%). (**B**) Eyes with EZ attenuation (*n* = 12) showed fewer and less localized microscotomas (3 eyes, 24%), primarily in temporal and inferior parafoveal regions. (**C**) Eyes with intact EZ (*n* = 33) exhibited only two temporal microscotomas (6%), with no defects detected in other sectors. This pattern demonstrates a strong but regionally variable relationship between EZ disruption and localized sensitivity loss, predominantly affecting the temporal MacTel zone

Microscotomas (< 10 dB) were identified in 10 eyes (18.5%), predominantly in the temporal macula (9 eyes). Figure 4 illustrates their distribution according to ellipsoid zone (EZ) status. Among eyes with EZ loss (*n* = 9), 55% (5 eyes) exhibited temporally located microscotomas, whereas 6% (2 eyes) of those with intact EZ (*n* = 33) showed similar findings, indicating a strong topographic association between EZ disruption and localized functional loss. Table 2 compares BCVA, CRT, and mRS among EZ status groups. LogMAR BCVA showed a stepwise worsening from EZ intact to EZ loss eyes and the difference was statistically significant after accounting for inter-eye correlation using linear mixed-effects models (0.11 ± 0.15, 0.23 ± 0.21, and 0.31 ± 0.27, respectively; *p* = 0.042). CRT, MD, and central retinal sensitivity did not differ significantly among EZ status groups.

**Table 2:** Comparison of retinal structure and function according to ellipsoid zone (EZ) integrity in macular telangiectasia type 2

	EZ intact (*n* = 33)	EZ irregular (*n* = 12)	EZ loss (*n* = 9)	*P* value
logMAR BCVA	0.11 ± 0.15	0.23 ± 0.21	0.31 ± 0.27	0.042
CRT (um)	261.81 ± 45.50	258.82 ± 50.71	226.56 ± 70.85	0.381
MD (dB)	1.06 ± 1.67	1.00 ± 1.87	-0.53 ± 2.09	0.147
Central RS (dB)	30.04 ± 4.81	28.64 ± 7.32	26.73 ± 2.23	0.293

Data are presented as mean ± standard deviation. *P* values were calculated using linear mixed-effects models

Statistically significant values are in bold

**Fig. 5:**
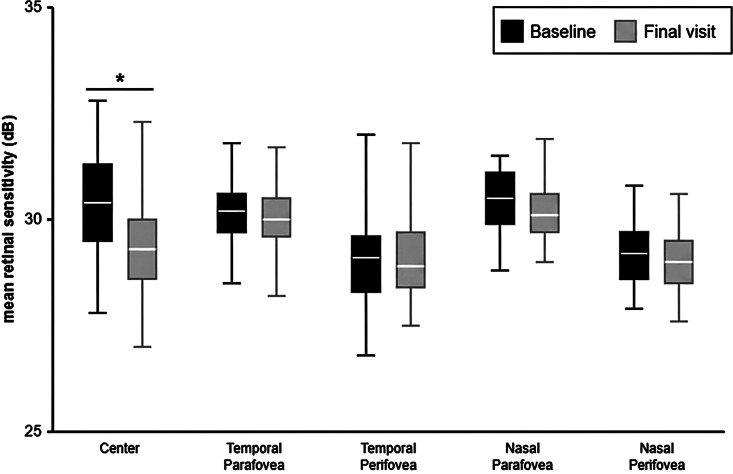
Longitudinal change in mean retinal sensitivity across macular subfields. Box-and-whisker plots show mean retinal sensitivity (mRS, dB) at baseline (black) and final visit (gray) across five ETDRS-derived macular regions: central, temporal parafovea, temporal perifovea, nasal parafovea, and nasal perifovea. In this analysis, parafovea and perifovea corresponded to the inner and outer ETDRS rings, respectively. A significant reduction in mRS was observed in the central subfield (*p < 0.05), whereas no significant changes were detected in the parafoveal or perifoveal regions. Boxes represent interquartile ranges, horizontal lines indicate medians, and whiskers denote the 10th–90th percentiles. This finding highlights selective central functional decline in macular telangiectasia type 2 despite stable parafoveal sensitivity

Longitudinal analysis included 38 eyes (mean follow-up 2.01 ± 1.10 years). No significant changes were observed in BCVA or retinal thickness across ETDRS subfields (Table 3). However, central mRS (F1) declined significantly (29.51 ± 2.02 dB to 28.84 ± 1.38 dB, *p* = 0.038), while temporal sectors remained stable. Notably, in all cases where a longitudinal increase in logMAR BCVA was observed, there was a concomitant reduction in central mean retinal sensitivity. This central sensitivity reduction (Fig. 5) suggests that Compass microperimetry may provide complementary functional information for longitudinal monitoring when BCVA and conventional OCT thickness parameters remain stable. On qualitative review of longitudinal OCT images, no clear categorical progression of EZ status was observed during follow-up.

**Table 3:** Longitudinal changes in retinal thickness and retinal sensitivity during follow-up in macular telangiectasia type 2

Parameters	Baseline	Final visit	*P* value
logMAR BCVA	0.14 ± 0.11	0.16 ± 0.12	0.261
**Retinal thickness** (um)			
Center	263.43 ± 79.30	260.32 ± 82.12	0.272
Temporal	287.84 ± 68.45	286.53 ± 72.15	0.181
Nasal	304.25 ± 80.41	305.41 ± 85.25	0.304
**Retinal sensitivity** (dB)			
MD	-0.27 ± 2.59	-0.57 ± 2.95	0.174
Center	29.51 ± 2.02	28.84 ± 1.38	**0.038**
Temporal perifovea	28.53 ± 3.07	28.41 ± 1.78	0.404
Temporal parafovea	29.56 ± 2.31	29.08 ± 1.51	0.298
Nasal perifovea	28.36 ± 1.86	28.59 ± 1.63	0.452
Nasal parafovea	29.90 ± 2.53	29.74 ± 2.51	0.511

Data are presented as mean ± standard deviation. *P* values were calculated using linear mixed-effects models

Statistically significant values are in bold

## Discussion

This study evaluated the topographic and longitudinal relationship between retinal sensitivity and OCT-based structural changes in macular telangiectasia type 2 (MacTel) using Compass fundus-tracked microperimetry. The main findings were that microscotomas were preferentially distributed in the temporal macula and were more frequent in eyes with EZ loss, while central mean retinal sensitivity showed a subtle longitudinal decline despite stable BCVA and conventional OCT thickness parameters over two years. These findings support the potential value of Compass microperimetry as a complementary functional measure in MacTel, particularly for detecting localized functional abnormalities that may not be reflected by BCVA or sectoral retinal thickness alone. The preferential thinning of the temporal retina and the spatial distribution of microscotomas mirrored MacTel’s well-known temporal predilection [17, 18].

These findings are biologically plausible in the context of MacTel pathophysiology. While MacTel was historically considered a vascular disorder, current evidence supports a primary neurodegenerative process involving Müller cell dysfunction, secondary photoreceptor compromise, and vascular remodeling [6, 12]. A localized reduction in retinal sensitivity may reflect impaired photoreceptor function or disturbed Müller cell–photoreceptor support before overt changes become apparent on conventional structural grading. However, this interpretation warrants caution, as subtle structural abnormalities may not be fully captured by qualitative OCT review.

Early structural changes in MacTel commonly include temporal parafoveal thinning and inner retinal cavitation [3]. Consistent with these features, ETDRS temporal subfields showed lower retinal thickness than nasal counterparts in our cohort. However, the relatively preserved baseline BCVA and retinal sensitivity indicate that our cohort was biased toward early or functionally preserved disease, likely because reliable microperimetry requires stable fixation and adequate test performance. This selection bias limits generalizability to advanced MacTel.

Our findings are consistent with previous MacTel Project studies demonstrating a close but imperfect structure–function relationship in MacTel. Vujosevic et al. showed that microperimetry-defined scotomas are often localized to the temporal parafoveal MacTel zone [13]. Heeren et al. demonstrated a longitudinal association between EZ loss on en face OCT and functional loss [12]. Sallo et al. similarly reported a robust correlation between EZ break area and microperimetry-based functional outcomes in a ciliary neurotrophic factor (CNTF) trial [7]. The present study does not replace these larger prospective or trial-based datasets; rather, it complements them by applying Compass fundus-tracked microperimetry with ETDRS sector–based spatial mapping in a real-world cohort.

Building on prior MP-1–based MacTel studies, microscotoma was defined as < 10 dB in the present study. Although retinal sensitivity values are not directly interchangeable across devices, Compass-based testing similarly showed scotomas localized to the temporal parafoveal MacTel zone, supporting the clinical relevance of this threshold in our cohort. Formal Compass-specific validation, however, remains necessary. Moreover, since Compass microperimeter provides more robust fundus-tracking and a wider dynamic range with reduced ceiling effects, these instrument characteristics might reduce the risk of over- or under-estimating scotoma borders, thereby enhancing the broader suitability of this device for future studies [19–21].

The most relevant longitudinal signal in this cohort was the selective decline in central mRS, which was observed despite stable BCVA and conventional OCT thickness parameters. This supports central mRS as a potentially sensitive functional readout for subtle change in early MacTel. However, this finding should not be interpreted as definitive evidence that functional loss precedes structural progression. Because structural assessment was limited to conventional OCT thickness parameters and qualitative outer retinal grading, subtle EZ enlargement or other structural alterations may not have been captured. In addition, the magnitude of central mRS decline was small and should be interpreted in light of known microperimetry test–retest variability in MacTel. Thus, central mRS may provide complementary longitudinal functional information, but its clinical significance at the individual-eye level requires further validation.

Strong association between outer retinal integrity assessed by ellipsoid zone status and microscotomas has been consistently reported, while acknowledging imperfect pointwise agreement at individual loci [22]. In addition, previous reports also showed that small breaks occasionally fell entirely between test points, yielding absent or underestimated functional loss despite definite structural damage [7]. Our data mirrored this pattern, 55% of eyes with EZ loss showing co-local microscotomas. Conversely, two eyes classified as having intact EZ demonstrated mild localized functional decline. These eyes exhibited a temporal microscotoma despite preserved BCVA and no definite EZ discontinuity on qualitative OCT review of foveal and parafoveal OCT B-scans. During follow-up, neither eye showed definite progression to EZ loss. These findings suggest that subtle functional abnormalities may be detectable before categorical qualitative EZ disruption becomes apparent, although subtle structural alterations below the resolution of qualitative grading cannot be excluded. In addition, the use of standard 10 − 2 grid and ETDRS sectoral averaging may have contributed to structure-function mismatch by undersampling or diluting subtle parafoveal defects. Looking ahead, MacTel-specific visual field grid with higher sampling density in the temporal parafovea may better characterize these structure-function relationships in early-stage disease [3].

The absence of clear categorical EZ progression should be interpreted in the context of the relatively short follow-up period and the qualitative nature of our OCT grading. Previous MacTel natural history studies have shown that EZ loss progresses slowly and non-linearly, with reported mean or median annual progression rates of approximately 0.057–0.08 mm²/year, depending on baseline lesion size and morphology [12, 22]. Therefore, subtle EZ enlargement over a 2-year period may not be captured by categorical OCT grading. Our findings should thus be interpreted as functional change detected despite stable BCVA, CRT, and qualitative EZ status, rather than definitive evidence that functional loss precedes all structural progression.

This study has several limitations. First, the retrospective design, modest sample size, and absence of a normal control group limit the generalizability of the findings. Second, although mixed-effects models were used to account for inter-eye correlation, the inclusion of both eyes from some patients remains a potential source of dependence. Third, the cohort may have been biased toward relatively early-stage MacTel because reliable microperimetry requires stable fixation and adequate test performance. Fourth, our ETDRS sector–based analysis improved interpretability but reduced spatial resolution and may have diluted localized parafoveal defects; pointwise structure–function analysis or MacTel-specific test grids would be more sensitive for detecting focal mismatch. Fifth, EZ progression was assessed qualitatively, and en face EZ loss area was not quantitatively measured; therefore, subtle structural progression could not be excluded. In addition, inner retinal parameters such as ganglion cell complex thickness were not quantitatively analyzed, and their potential contribution to retinal sensitivity loss should be evaluated in future studies. Finally, the microscotoma threshold of < 10 dB was adopted from prior microperimetry literature, but device-specific validation for Compass microperimetry in MacTel remains limited.

In conclusion, Compass fundus-tracked microperimetry demonstrated localized functional abnormalities that broadly corresponded to EZ integrity in MacTel type 2, while also showing occasional structure–function mismatch. Central mRS may provide complementary functional information when BCVA and conventional OCT parameters remain stable. Larger prospective studies using quantitative EZ area measurement, pointwise structure–function analysis, and MacTel-specific microperimetry grids are warranted to validate its role as a progression marker and trial endpoint.

## Data Availability

The datasets used and/or analyzed during this study can be made available by the corresponding author upon reasonable request.
